# From high-throughput evaluation to wet-lab studies: advancing mutation effect prediction with a retrieval-enhanced model

**DOI:** 10.1093/bioinformatics/btaf189

**Published:** 2025-07-15

**Authors:** Yang Tan, Ruilin Wang, Banghao Wu, Liang Hong, Bingxin Zhou

**Affiliations:** Institute of Natural Sciences, Shanghai Jiao Tong University, Shanghai, 200240, China; School of Information and Science, East China University of Science and Technology, Shanghai, 200231, China; Shanghai Artificial Intelligence Laboratory, Shanghai, 200232, China; Zhangjiang Institute for Advanced Study, Shanghai Jiao Tong University, Shanghai, 201203, China; School of Information and Science, East China University of Science and Technology, Shanghai, 200231, China; Institute of Natural Sciences, Shanghai Jiao Tong University, Shanghai, 200240, China; Zhangjiang Institute for Advanced Study, Shanghai Jiao Tong University, Shanghai, 201203, China; Institute of Natural Sciences, Shanghai Jiao Tong University, Shanghai, 200240, China; Shanghai Artificial Intelligence Laboratory, Shanghai, 200232, China; Zhangjiang Institute for Advanced Study, Shanghai Jiao Tong University, Shanghai, 201203, China; Institute of Natural Sciences, Shanghai Jiao Tong University, Shanghai, 200240, China; Zhangjiang Institute for Advanced Study, Shanghai Jiao Tong University, Shanghai, 201203, China

## Abstract

**Motivation:**

Enzyme engineering is a critical approach for producing enzymes that meet industrial and research demands by modifying wild-type proteins to enhance properties such as catalytic activity and thermostability. Beyond traditional directed evolution and rational design, recent advancements in deep learning offer cost-effective and high-performance alternatives. By encoding implicit coevolutionary patterns, these pretrained models have become powerful tools, with the central challenge being to uncover the intricate relationships among protein sequence, structure, and function.

**Results:**

We present VenusREM, a retrieval-enhanced protein language model designed to capture local amino acid interactions in both spatial and temporal scales. VenusREM achieves state-of-the-art performance on 217 assays from the ProteinGym benchmark. Beyond high-throughput open benchmark validations, we conducted a low-throughput post hoc analysis on more than 30 mutants to verify the model’s ability to improve the stability and binding affinity of a VHH antibody. We also validated the effectiveness of VenusREM by designing 10 novel mutants of a DNA polymerase and performing wet-lab experiments to evaluate their enhanced activity at elevated temperatures. Both *in silico* and experimental evaluations not only confirm the reliability of VenusREM as a computational tool for enzyme engineering but also demonstrate a comprehensive evaluation framework for future computational studies in mutation effect prediction.

**Availability and implementation:**

The implementation is available at https://github.com/tyang816/VenusREM.

## 1 Introduction

Enzymes are fundamental components of synthetic biology systems. Wild-type enzymes often face limitations such as low catalytic activity, poor stability, and insufficient binding affinity, which restrict their applications in both academic research and industrial practice (Zhou *et al.* 2024a). Through enzyme engineering, wild-type proteins can be modified to enhance these properties, enabling them to meet the requirements of specific applications (Lu *et al.* 2022, Zhou *et al.* 2024b).

With the rapid expansion of protein databases and continuous advancements in artificial intelligence, deep learning methods offer new possibilities for enzyme engineering. Typically, a deep neural network for proteins is pretrained on large-scale protein sequence data (with optional structural information) to learn to extract numerical representations of proteins, where the resulting embeddings are then used to score and rank candidate mutants (Meier *et al.* 2021, Notin *et al.* 2022a, Lin *et al.* 2023). This prediction task, known as *mutation effect prediction*, is often evaluated using high-throughput datasets from deep mutation scanning (DMS), e.g. ProteinGym (Notin *et al.* 2024).

Existing pretraining schemes can be broadly divided into three categories: sequence-based, structure-based, and evolution-based approaches. Sequence-based methods are the most popular choice. They analyze the implicit pairwise relationships between amino acids (AAs) to learn protein sequence representations. The objective of pretraining such a protein language model (PLM) is to predict unknown AA types in a sequence from partial input, typically by autoregressively generating AAs along the sequence index (Notin *et al.* 2022b, Madani *et al.* 2023) or by randomly masking a set of AAs and recovering them (Rives *et al.* 2021, Li *et al.* 2024). The second category of structure-based methods incorporates topological inductive biases to capture stronger interactions between spatially adjacent AAs (Hsu *et al.* 2022, Zhou *et al.* 2024c). Geometric deep learning methods are typically applied to embed these associated structural constraints. The third category introduces multiple sequence alignment (MSA) data into the model to provide evolutionary information about proteins (Notin *et al.* 2022a, Roshan et al. 2021). MSA reflects protein conservation and the variation patterns occurring through natural evolution. Although lacking explicit functional labels, homologous sequences offer valuable information beyond a single sequence or structure for predicting protein assays.

A natural question that arises when constructing implicit representations for proteins using deep learning is: *how can we effectively integrate the nonorthogonal sequence, structure, and evolutionary characteristics?* Although some works have considered encoding two types of information simultaneously, such as sequence and MSA information (Roshan *et al.* 2021) or sequence and structure information (Tan *et al.* 2023, 2024a, Yang *et al.* 2023), to the best of our knowledge, no work has yet integrated all three types. Also, when incorporating homology information, existing methods either require additional training procedures (e.g. EVE; Frazer *et al.* 2021) or lack plug-and-play flexibility (e.g. MSA transformer; Roshan *et al.* 2021) and PoET; Truong and Bepler 2023; both include evolutionary information with model-specific designs).

**Figure 1.**
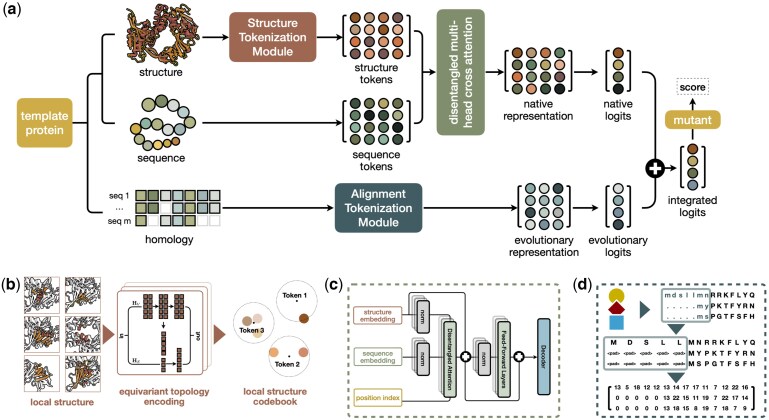
Workflow of VenusREM for predicting mutation effects. (a) For a given template protein, VenusREM encodes structural, sequence, and MSA information to generate logits for each residue, which are used to calculate mutation fitness scores. (b) For each AA, its local structure is clustered into 2048 distinct structure tokens. (c) The vector representations of structural and sequence information are integrated using disentangled cross-attention through BERT-style pretraining. (d) Homologous information is retrieved via Jackhmmer and converted to a matrix representation of evolutionary logits.

Consequently, protein representations learned from pretraining frameworks may lack a piece of crucial features that could significantly impact function. To address this gap, we propose a new pretrained PLM with a *R*etrieval-*E*nhanced *M*odule, namely VenusREM (Fig. 1). We design the model to uncover implicit representations of native features based on sequence and structure tokens, where sequence–structure embeddings are integrated through disentangled multihead cross-attention layers trained in a BERT-style manner. The evolutionary representations are determined by an alignment tokenization module without additional training workload and are integrated into the fitness evaluation of mutants in a plug-and-play fashion.

To validate the effectiveness of VenusREM, we examine two scenarios: (i) *Can VenusREM consistently outperform the prediction performance of previous methods across diverse proteins and assays on high-throughput experimental data* (which is more comprehensive and contains more records)? (ii) *Can VenusREM provide reliable predictions to help biologists identify expected single-site and multisite mutation designs in practice* (i.e. low-throughput experiments with more precise quantitative measurements)? To address the first question, we evaluate Spearman’s ρ between predicted mutation fitness and experimental scores on over 2 million mutants from 217 assays in ProteinGym, the largest open benchmark, and compare VenusREM with all models on the public leaderboard. The superior performance of our model across all functions and properties underscores its leading position in general-purpose mutation effect prediction. For the second question, we conduct both *in silico* analysis on the variable domain of the heavy chain of a nano-antibody targeting growth hormone (VHH antibody) and experimental evaluation on the bacteriophage phi29 DNA polymerase (phi29 DNAP). We discuss the model’s feasibility in enhancing various assays (e.g. stability, activity, and binding affinity) with both single-site and multisites mutations. VenusREM effectively identify positive mutations and rank mutant performance scores, demonstrating significant potential for identifying highly active and stable mutants from the complete search space, thereby providing pivotal support for enhancing properties in enzyme engineering.

## 2 Materials and methods

This section overviews the development and evaluation of VenusREM. The overall architecture of VenusREM is first introduced to establish a comprehensive understanding of its design principles. Next, we detail the preparation of input data, with a particular emphasis on sequence and structure homology searches. We then describe the computational methods for integrating multimodal information, including the design of the cross-attention module to unify sequence and structural features, and strategies for retrieving and incorporating homologous sequence data. Lastly, we outline the experimental evaluation protocols of both *in silico* experiments and wet-lab assays.

### 2.1 Model overview

For a given template protein, VenusREM receives three sets of inputs: sequence, structure, and homolog information (Fig. 1a). The sequence and structure inputs are tokenized and processed by a pretrained PLM to generate a native representation of the single protein (Fig. 1b and c). Simultaneously, homologous family sequences—identified based on sequence and/or structural similarity to the template protein—are summarized and integrated into the model (Fig. 1d). Both the native representation and the evolutionary representations are vectorized descriptions of AA positions and types. These representations are further processed through a specifically defined scoring rule to compute the final mutation fitness score.

### 2.2 Data preparation

#### 2.2.1 Sequence and structure tokens

We first extract discrete representations of the sequence and structure, which are used as inputs for the PLM to generate vector representations. The sequence input is inherently discrete and can be tokenized directly without additional preprocessing. For the local structure of AAs, we utilize a *structure tokenization module*, which constructs a codebook with 2048 dimensions to encode structural information effectively. Both sequence and structure tokens will later be sent to disentangled multihead attention to encode native representation.

##### 2.2.1.1 Residue-level sequence tokenization

The construction of sequence tokens is relatively straightforward. We construct a vocabulary for 20 standard AAs and 5 special characters (i.e. <pad> , <cls> , <eos> , <unk>, and <mask>) to encode the AA sequence. Each sequence of L AAs is mapped to a one-hot vector of size L×25. The training set for the pretrained language model is from Foldseek, which clusters the AlphaFold2 database to retain the original PDB structures and AA sequences. For inference, only the characteristics of the wild-type protein (sequence and structure) are required as input for tokenization and homolog sequence retrieval. Unlike other approaches, our method does not rely on mutating each position according to the DMS data (Notin *et al.* 2022a) or applying mask operations (Su *et al.* 2023) during input preparation.

##### 2.2.1.2 Local structure tokenization

Following Li *et al.* (2024) and Tan *et al.* (2024b), we represent local protein structures by a codebook of discrete structural representations on a per-residue basis. Finding token representations for local protein structures involves two key steps, including local structure extraction and latent structure vocabulary assignment (Fig. 1b). Consider a protein with L AAs. For each residue, we define its local structure by considering up to 40 neighboring residues within a 10 Å spatial radius. We defined an undirected graph with each node representing a residue, and two nodes are connected if their spatial distances are <10 Å. The constructed L graphs are then fed into pretrained geometric vector perceptrons (GVP) (Jing *et al.* 2021), denote as $\pi_{\theta }(G)\in R^{L\times 256}$. The GVP is integrated with a decoder to form an autoencoder, trained with a denoising pretraining objective by perturbing $C_{\alpha }$ coordinates with 3D Gaussian noise and applying Brownian motion. We train a six-layer GVP with 4, 735, 677 graphs of local structures from **CATH43-S40** (Orengo *et al.* 1997). After obtaining the matrix representation, a mean pooling operation is then applied to produce the final vector representation of local structures. These 256-dimensional vectors in the continuous space are further mapped to a 2048-dimensional discrete space using K-means clustering for a balance between efficiency and representativeness, where each dimension represents an implicit vocabulary of structures.

#### 2.2.2 Homological sequences

In addition to the sequence- and structure-based native information, VenusREM incorporates evolutionary information retrieved from homologous sequences. We consider three retrieval strategies, each tailored to leverage distinct sources and formats of alignment data.

##### 2.2.2.1 EVcouplings alignment

For each assay in ProteinGym, we utilized the curated MSAs provided by the official source. In cases where domain information was available, alignments were restricted to the domain regions. Homologous sequences were retrieved using Jackhmmer (Eddy 2011), a profile hidden Markov model-based tool, with five iterations and a bit score ranging from 0.1 to 0.9, resulting in nine distinct search groups, with searches performed on the Uniref100 dataset (downloaded on 24 November 2021). Subsequently, the EVcouplings (Hopf *et al.* 2014) library was employed to select the .a2m file containing the highest number of significant evolutionary couplings among the retrieved alignments. To prepare the final alignment files, lowercase letters in the .a2m files were converted to uppercase, and special characters were replaced with the <pad> token. This preprocessing ensured the alignments were suitable for downstream analyses.

##### 2.2.2.2 ColabFold alignment

The second strategy uses ColabFold (Mirdita *et al.* 2022) to automate MSA retrieval by querying a local PDB database, which outputs alignments in the .a3m format. Unlike .a2m by EVcouplings, the .a3m format compresses gaps in the query sequence to enhance computational efficiency. It is notable that the .a3m files generated by ColabFold are not fully aligned. To address this, we apply the reformat.pl script to adjust and realign the sequences, which ensures compatibility with downstream analyses. This integrated approach combines automated retrieval and alignment refinement, thus optimizes both computational performance and the accuracy of MSAs.

##### 2.2.2.3 Foldseek alignment

In the third strategy, structural alignment data are retrieved with efficient implementation by the Foldseek API (Van Kempen *et al.* 2024). Queries could be submitted via HTTP POST requests, targeting multiple protein structure databases, such as afdb50, afdb-proteome, cath50, and pdb100 (detailed in Supplementary Table S3). In short, the pipeline begins with uploading the query structure (.pdb) and monitoring the server response in a loop until the search status is marked as “COMPLETE”. The result ticket is extracted during each iteration, with the .json response parsed to confirm progress. In the event of errors, the system retries after a brief delay. Upon receiving the final results, the alignment data is processed to extract meaningful information. Specifically, the query and target alignments are parsed from the results, with gaps removed from the query sequence and the target sequence adjusted accordingly. To ensure consistency, the final target alignment is padded with gaps to match the length of the query sequence. The processed alignments are then stored in a dictionary, indexed by key target information, including sequence name, probability, evaluation score, and alignment positions.

We tested the three homology searching strategies in this study (Supplementary Table S2). In the case of the 217 assays in ProteinGym, the corresponding .a2m documents used in the first strategy are directly downloaded from https://marks.hms.harvard.edu/proteingym/DMS_msa_files.zip. For the other two search strategies (based on ColabFold and Foldseek), we search for homologous sequences according to the protocol we have introduced above. The additional computational steps for summarizing evolutionary representations from these phonological sequences will be introduced in the next section.

### 2.3 Model construction

#### 2.3.1 Native representation calculation

The native representation, i.e. the joint embeddings of protein sequences and structures, are obtained by a BERT-style PLM. The core propagation rule is based on disentangled multihead cross-attention (He *et al.* 2020, Li *et al.* 2024) (Fig. 1c). For a protein of length L, we define the token inputs for its AA sequence and structure as R and S. For two arbitrary AAs at positions i and j, their attention score Attn(i,j) is calculated based on R, S, and their relative position P:


(1) $$\text{Attn}(i,j)=\{R_{i},S_{i},P_{ij}\}\times {\left\{R_{j},S_{j},P_{ji}\right\}}^{⊤},$$

where $P_{ij}$ represents the relative position from the ith AA to the jth AA. After the element-wise multiplication in (1), We further simplify the equation by discarding terms that do not contain residue information, i.e. $S_{j}S_{j}^{⊤},S_{i}P_{ji}^{⊤},P_{ji}S_{j}^{⊤},P_{ji}P_{ji}^{⊤}$, and retain only the five AA-relevant attention scores and rewrite them with the query, key, and value matrices, which are obtained via linear transformations during forward propagation:


(2) $$\begin{array}{l} \text{Attn}(i,j)=R_{i}R_{j}^{⊤}+R_{i}S_{j}^{⊤}+R_{i}P_{ji}^{⊤}+S_{i}R_{j}^{⊤}+P_{ij}R_{j}^{⊤}\\ =Q_{i}^{R}{\left(K_{j}^{R}\right)}^{⊤}+Q_{i}^{R}{\left(K_{j}^{S}\right)}^{⊤}+Q_{i}^{R}{\left(K_{ji}^{P}\right)}^{⊤}\\ \begin{array}{c} \;+Q_{i}^{S}{\left(K_{j}^{R}\right)}^{⊤}+Q_{ij}^{P}{\left(K_{j}^{R}\right)}^{⊤}. \end{array} \end{array}$$

The notations follows conventional attention. The subscription denotes their association with the AA and the superscription denotes their association to the input, e.g. $Q_{i}^{R}$ represents the query value for the ith AA on its sequence information and $K_{ij}^{P}$ represents the key value with respect to the relative distance from the ith AA to the jth AA.

Similar to classic attention schemes, the initial attention scores obtained from (Native Representation Calculation) require element-wise normalization by a scaling factor $1/\sqrt{5d}$ with d being the dimension of $Q^{R}$. Denote the unnormalized attention matrix as $H_{\text{attn}}$. We use it to update the hidden representation for R, which reads:


(3) $$R_{o}=\sigma (\frac{H_{\text{attn}}}{\sqrt{5d}})V^{R},$$

where σ(·) is a softmax activation function which operates in the last dimension and $V^{R}$ is the value matrix of R.

#### 2.3.2 Evolutionary representation calculation

We process homologous sequences to extract evolutionary representations. For simplicity, we replace the gap character—with a special token <pad>. Suppose N homologous sequences are found for a protein of length L, denoted by $A\in Z^{N\times L}$. A counting matrix $C\in R^{L\times V}$ records the frequencies of AA types at each residue position, where


(4) $$C_{iv}=\frac{\sum_{n=1}^{N}I(A_{ni}=v)}{\sum_{v=1}^{V}\sum_{n=1}^{N}I(A_{ni}=v)}.$$

The vocabulary size V=25 accounts for 20 AAs and 5 special tokens. The indicator function $I(A_{ni}=v)$ assigns 1 if the ith position (1≤i≤L) of the nth sequence (1≤n≤N) fills the vth token (1≤v≤V), otherwise 0. The final evolutionary logits $O^{\text{evo}}$ are generated from C with subsequent normalization and log-transformation, i.e.


(5) $$O_{iv}^{\text{evo}}=\text{log}\;\left(\frac{\;\text{exp}(C_{iv})}{\sum_{v=1}^{V}\;\text{exp}\;(C_{iv})}\right).$$

#### 2.3.3 Zero-shot fitness scoring

For a given mutant, its fitness score is calculated by comparing the predicted logits of the mutant residues with those of the wild-type residues. We first predict the native logits $O_{iv}^{\text{native}}$ from the pretrained PLM, and combines it with evolutionary logits $O_{iv}^{\text{evo}}$:


(6) $$O_{iv}^{\text{out}}=(1-\alpha )\cdot O_{iv}^{\text{native}}+\alpha \cdot O_{iv}^{\text{evo}},$$

where the retrieval ratio α∈[0,1] controls the weight of integrating the intrinsic and evolution probability distributions.

The mutation fitness scores are calculated from $O^{\text{out}}$. For a mutant x, the overall fitness score $F_{x}$ is obtained by summing the associated logit differences across all mutation sites t∈T:


(7) $$F_{x}=\sum_{t\in T}\left(O_{tv^{\prime }}^{\text{out}}-O_{tv}^{\text{out}}\right),$$

where at position t, the mutant alters the residue type from the wild-type v to mutate residue $v^{\prime }$. This scoring method captures the relative fitness by evaluating how much the mutation alters the predicted logit values compared to the wild type, with larger logit differences reflecting more significant deviations in fitness. For multiple mutations, the logit differences are summed over all mutated sites to reflect the cumulative effect of all alterations.

### 2.4 *In silico* evaluation

#### 2.4.1 Baseline methods

On the high-throughput public benchmark dataset ProteinGym, we compared VenusREM against a range of baseline zero-shot protein models, including sequence-based models, sequence-structure models, alignment-based models, and inverse folding models. We selected the top 50 models listed on the ProteinGym leaderboard (https://proteingym.org/benchmarks) as of the submission deadline. For clarity, we chose the best-performing version of each model based on the overall score. All models were evaluated under ProteinGym’s standardized protocol, which includes calculating Spearman’s correlation coefficients and the corresponding standard deviation. All baseline methods are open-sourced with readily available checkpoints for direct implementation (Supplementary Table S1).

For the case study comparisons, we compare the performance of VenusREM with leading sequence-based (ESM2 and ESM-1b) and sequence–structure models (SaProt, ProtSSN, and MIF-ST) to highlight its advantages.

#### 2.4.2 Experimental protocol

The official implementation of VenusREM is at https://github.com/tyang816/VenusREM. The model training and inference are performed on four NVIDIA 3080 GPUs with 10GB VRAM, using PyTorch version 2.4.1. The Transformers library version 4.45.0 is used for the PLM module. We freeze the pretrained parameters for native representation calculation available at https://huggingface.co/AI4Protein/ProSST-2048. Other environment configurations for reproducing the model can be found in the GitHub repository. Homology sequence searches are conducted using the Conda-maintained Jackhmmer and EVcouplings libraries, with an additional plmc dependency required for EVcouplings. We set the optimal setting for the retrieval factor α=0.8.

#### 2.4.3 Benchmark datasets

##### 2.4.3.1 ProteinGym: high-throughput evaluation

ProteinGym is a comprehensive suite of benchmarks designed to evaluate the proficiency of models in forecasting the impacts of protein mutations. These benchmarks are categorized based on the nature of the mutations (substitutions versus indels) and the origin of the ground truth data (DMS assay versus clinical annotation). In our study, we have opted to utilize the substitution mutations to demonstrate the capabilities of our model. This subset encompasses 217 high-throughput assays, totaling more than 2.5 million mutations.

##### 2.4.3.2 VHH antibody: post hoc analysis with existing experimental results

We extracted the experimental data of 31 VHH antibody mutants from Kang *et al.* (2025), covering 1–4 site mutations for binding affinity and alkali resistance. For both assays, we used the average EC50 values from three repeated experiments. The wild-type VHH antibody sequence is as follows:

MQVQLVESGGGLAQAGGSLRLSCAVSGMPEFARAMGWFRQAPGKERELLAAIEGIGATTYYADSVKGRFTISRDDAANTVLLQMNSLKPDDTAVYYCAAAFSVTIPTRARHWVDWGPGTLVTVSSDDDDKSGGGGSHHHHHH

The corresponding structure is predicted by AlphaFold3 server (Abramson *et al.* 2024). The homology alignment sequences are searched from UniRef100 database (downloaded in October 2024).

### 2.5 Wet-lab examination

We followed the same approach as in the previous experiments on the VHH antibody, using VenusREM to evaluate phi29 DNA polymerase’s C3 mutant as the starting point. Single-point saturation mutations were scored, and the top 10 mutants with the highest scores were selected for experimental validation. These experiments assessed the activity and thermostability of the selected mutants compared to the C3 mutant. The sequence of the C3 mutant is provided below:

MKHMPRKRYSCDFETTTKVEDCRVWAYGYMNIEDHSEYKIGNSLDEFMAWALKVQADLYFHNLKFDGAFIINWLERNGFKWSADGLPNTYNTIISRMGQWYMIDICLGYKGKRKIHTVIYDSLKKLPFPVKKIAKDFKLTVLKGDIDYHKERPVGYKITPEEYAYIKNDIQIIAEALLIQFKQGLDRMTAGSDSLKDFKDIITTKKFKKVFPTLSLELDKKVRYAYRGGFTWLNDRFKEKEIGEGMVFDVNSLYPAQMYSRLLPYGEPIVFEGKYVWDEDYPLHIQHIRCEFELKEGYIPTIQIKRSRFYKGNEYLKSSGGEIADLWLSNVDLELMKEHYDLYNVEYISGLKFKATTGLFKDFIDKWTYIKTTSEGAIKQLAKLMLNSLYGKFASNPDVTGKVPYLKENGALGFRLGEEETKDPVYTPMGVFITAWARYTTITAAQACYDRIIYCDTDSIHLTGTEIPDVIKDKVDPKKLGYWAHESTFKRAKYLRPKTYIQDIYMKEVDGELVEGSPDDYTDIKFSVKCAGMTDKIKKEVTFENFKVGFSRKMKPKPVQVPGGVVLVDDTFTIK.

The detailed experimental procedures are provided in the Supplementary Material.

## 3 Results

This section presents *in silico* analyses and experimental assessments to comprehensively evaluate the performance of VenusREM across various properties and protein types. The following content highlights VenusREM’s performance on 217 DMS protein assays with over 2 million records. We also extract low-throughput experimental results from previous studies to assess the model’s reliability in predicting multi-site mutants for specific proteins. Additionally, we used VenusREM to engineer phi29 DNAP and experimentally validated the activity and thermostability on 10 single-site mutants.

### 3.1 VenusREM ranks top on ProteinGym leaderboard with significant performance improvement

The first experiment assesses the model’s performance on high-throughput open benchmarks. We conducted predictive evaluations using the testing data and procedures provided in the official ProteinGym repository (https://github.com/OATML-Markslab/ProteinGym). For each of the 217 assays, we used the sequence, structure (predicted by ColabFold 1.5; Mirdita *et al.* 2022), and homology sequences (retrieved by EVcouplings; Hopf *et al.* 2014) provided in the repository. For each DMS dataset, the model predicts the fitness scores of the mutants and calculates the weighted average Spearman’s ρ correlation between the predicted and ground truth scores. The standard deviation evaluates the stability of the model, which was computed using the bootstrap method defined in the official test script. We compared the overall performance of VenusREM with the top 18 models on the leaderboard. To avoid repetition, we retained only the highest scoring version for each method and reported their ranks in the full leaderboard in the first column of the table. The complete leaderboard is at https://proteingym.org/benchmarks.

**Figure 2.**
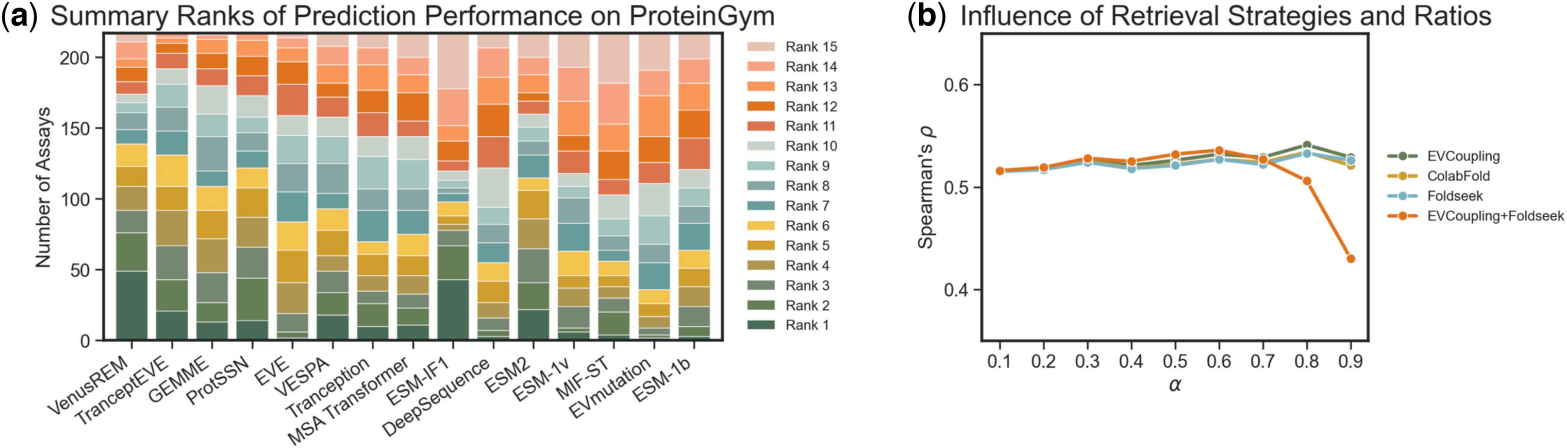
A summary of baseline comparisons on the ProteinGym mutation effect prediction task. (a) Performance ranks across each assay. for instance, a Rank 1 for VenusREM with a value of 49 indicates that VenusREM achieves the highest performance on 49 out of 217 assays. (b) Performance of VenusREM’s ablation models with various homologous sequence search strategies and retrieval ratios, assessed on a 10% randomly split validation set.

As reported in Table 1 and Supplementary Table S4, our method demonstrates significant improvements over baseline methods in both overall and property-specific evaluations. Previous studies (Tan *et al.* 2023, Notin *et al.* 2024) found that structure-aware models (e.g. SaProt, ProtSSN, and ESM-if1) are considered to perform better in binding and stability predictions, while evolution-aware models (e.g. TranceptEVE, MSA Transformer) tend to achieve higher scores in activity prediction. In response, VenusREM integrates sequence, structure, and evolutionary information to deliver the best overall performance and consistently ranks at the top across different individual property types. A summary ranking of individual predictions among all baseline methods is shown in Fig. 2a, with individual scores detailed in Supplementary Figs S3–S7. In terms of the number of assays where the best rank is achieved, VenusREM outperforms all other baseline methods with the highest top-rank count (e.g. top 3, shown by the green bars) and the fewest lower rank count (e.g. ranks after 10, shown by the red bars). We also examined VenusREM’s performance with different homology sequence retrieval strategies (Fig. 2b). The validation scores were assessed on a subset of ProteinGym with 10% random samples from each of the 217 assays. Overall, incorporating either sequence or structural homologous sequences improves the model’s fitness prediction performance, and they are considerably insensitive to the choice of retrieval ratio [defined in (6)], with validation scores remaining stable between 0.51 and 0.54. The highest performance for all methods occurs at ratios of 0.8 and 0.6. Based on the ablation study results on the validation set (Supplementary Table S5), we set the default retrieval method for VenusREM to EVcouplings with a 0.8 retrieval ratio.

**Table 1.** Spearman’s ρ correlation of mutation effect prediction (substitution) by zero-shot predictions on ProteinGym of different functions.

Rank	Model	seq	str	evo	Avg. Spearman	Activity	Binding	Expression	Organismal	Stability
	VenusREM	√	√	√	0.518±0.000^a^	0.499^a^	0.454^a^	0.533^a^	0.455^c^	0.649^b^
1	ProSST ([Li *et al.* 2024)	√	√		0.507±0.000^b^	0.476	0.445^b^	0.530^b^	0.431	0.653^a^
5	PoET (Truong and Bepler 2023)	√	√		0.470±0.009^c^	0.494^b^	0.396^c^	0.466	0.475^a^	0.519
7	VespaG (Marquet *et al.* 2024)	√	√		0.458±0.008	0.493^c^	0.370	0.456	0.437	0.533
8	SaProt (Su *et al.* 2023)	√	√		0.457±0.009	0.458	0.378	0.488^c^	0.367	0.592
9	TranceptEVE (Notin *et al.* 2022a)	√		√	0.456±0.009	0.487	0.376	0.457	0.460^b^	0.500
10	GEMME (Laine *et al.* 2019)			√	0.455±0.012	0.482	0.383	0.438	0.452	0.519
13	ProtSSN (Tan *et al.* 2023)	√	√		0.449±0.008	0.466	0.366	0.449	0.396	0.568
16	EVE (Frazer *et al.* 2021)			√	0.439±0.012	0.464	0.386	0.408	0.447	0.491
22	VESPA (Marquet *et al.* 2022)	√			0.436±0.008	0.468	0.366	0.404	0.440	0.500
23	Tranception (Notin *et al.* 2022b)	√		√	0.434±0.009	0.465	0.349	0.450	0.436	0.471
26	MSA Trans (Roshan *et al.* 2021)	√		√	0.432±0.011	0.469	0.337	0.446	0.421	0.495
29	ESM-if1 (Hsu *et al.* 2022)		√		0.422±0.013	0.368	0.389	0.407	0.324	0.624^c^
31	DeepSequence (Riesselman *et al.* 2018)			√	0.419±0.013	0.455	0.363	0.390	0.413	0.476
34	ESM2 (Lin *et al.* 2023)	√			0.414±0.014	0.425	0.337	0.415	0.369	0.523
36	ESM-1v (Meier *et al.* 2021)	√			0.407±0.015	0.420	0.320	0.429	0.387	0.477
40	MIF-ST (Yang *et al.* 2023)	√	√		0.400±0.011	0.390	0.321	0.438	0.366	0.485
41	EVmutation (Hopf *et al.* 2017]			√	0.395±0.010	0.440	0.317	0.378	0.411	0.430
42	ESM-1b [Rives *et al.* 2021)	√			0.394±0.013	0.428	0.287	0.406	0.351	0.500

a Score of the best-performing model.

b Score of the second-best-performing model.

c Score of the third-best-performing model.

### 3.2 VenusREM performs reliable prediction on favorable alkali resistance and binding affinity of VHH mutants

The second evaluation validates VenusREM’s effectiveness in fitness prediction for both single-site and multisite mutants. The engineering target is to enhance binding affinity and alkali resistance in the VHH antibody. This antibody type plays a crucial role in the development of clinical antibody-based therapeutics, serving as an affinity ligand to selectively purify biopharmaceuticals (Wang *et al.* 2014). In production, a clean-in-place process is typically required, which involves cleaning with an extremely alkaline solution for 24 h to eliminate impurities and contaminants. Therefore, improving the binding affinity and alkali resistance of the VHH antibody is a key requirement for industrial production. Here, we used experimental data from Kang *et al.* (2025) on 31 VHH antibody mutants with modifications at 1–4 sites. Their binding affinity and alkali resistance were assessed by EC50 values before and after treatment with 0.5 M NaOH for 24 h.

**Figure 3.**
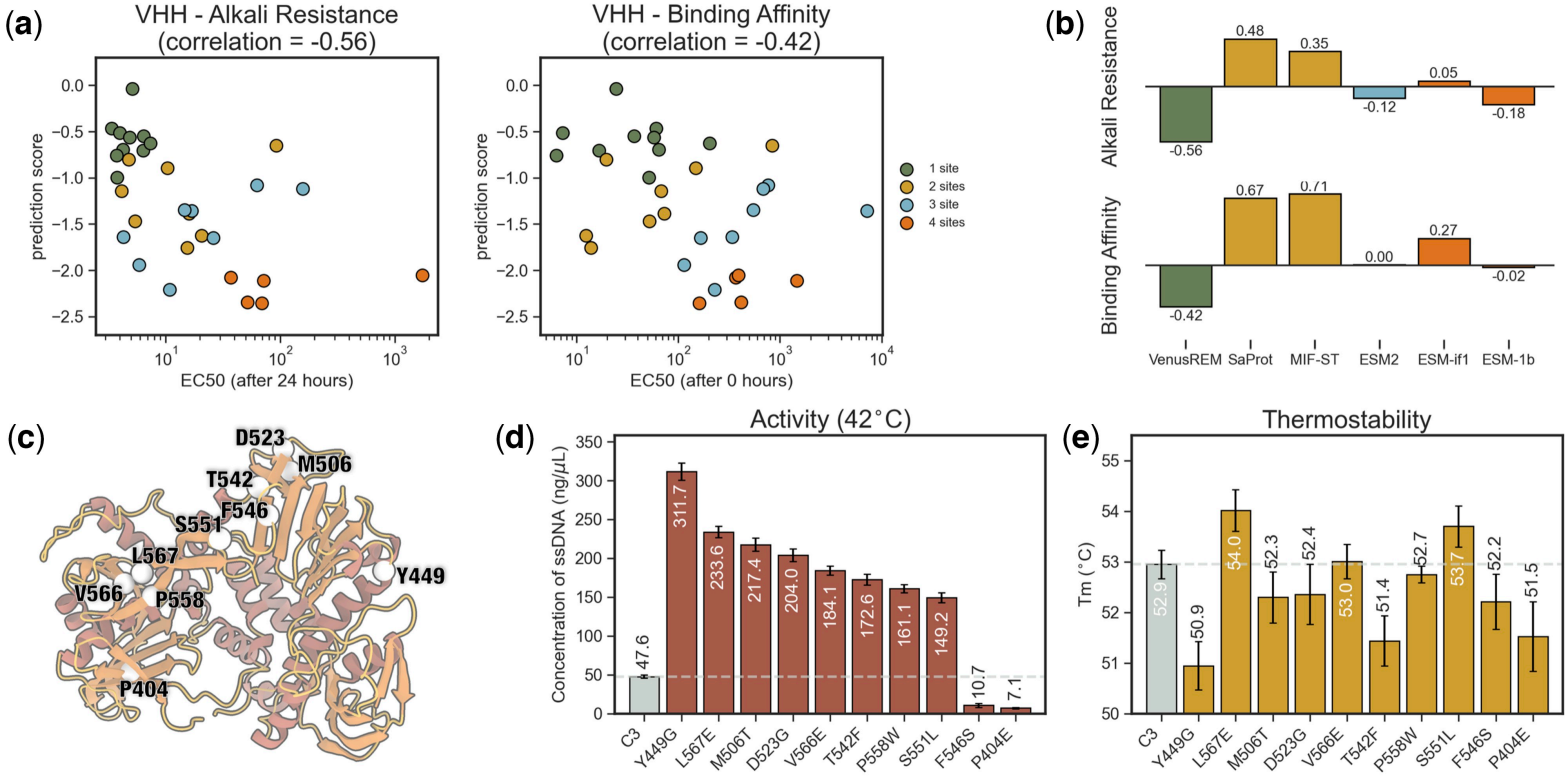
Performance analysis on low-throughput experimental datasets. (a) Scatter plot of predicted fitness scores (by VenusREM) versus experimentally obtained EC50 values. For both alkali resistance and binding affinity improvements, VenusREM’s scoring of 31 VHH antibody mutants by 1–4 sites shows a clear correlation with experimental data. (b) Performance of different models on the two assays of VHH antibody data. Only VenusREM successfully generated fitness scores that are moderately negatively correlated with EC50 values. (c) 3D structure of the template phi29 DNAP. The AA sites targeted for mutation across the 10 single-site mutants are highlighted and labeled with their wild-type residues. (d) Activity improvements in phi29 DNAP mutants. Among the 10 single-site mutants experimentally tested, 8 shows significant activity enhancements, with the top mutant exhibiting an eight-fold increase. (e) Thermostability of phi29 DNAP mutants. Three mutants demonstrate improvements in both thermostability and activity, with two of them showing significant gains.

Figure 3a visualizes VenusREM’s fitness prediction scores with respect to experimental results (retrieved from Kang *et al.* (2025). Here, each point represents a mutant, and the gray pentagon denotes the wild-type template. Mutants are color-coded based on mutation depth. Overall, there is a significant correlation between the fitness prediction scores and experimental results. Note that higher EC50 values indicate poorer binding affinity and alkali resistance for VHH. Thus, a correlation closer to −1 represents better fitness prediction. In Fig. 3b, we also compare VenusREM with other popular sequence- and structure-based baseline methods with their top-performing versions from ProteinGym. Results indicate that most models fail to establish a negative correlation between fitness and the two experimental indicators. Although ESM2 and ESM1b achieve a negative correlation, the values remain close to zero, suggesting limited practical utility for these models in engineering the VHH antibody.

### 3.3 VenusREM engineers phi29 DNAP toward significant activity enhancement with high positive rate

In the third experiment, we used VenusREM to enhance the catalytic activity of phi29 DNAP at higher temperatures. phi29 DNAP plays a key role in advancing isothermal amplification methods such as multiple displacement amplification and rolling-circle amplification (RCA) (Povilaitis *et al.* 2016). It facilitates efficient DNA amplification with low error rates, due to its remarkable strand-displacing activity with high fidelity and transcription processivity. While the existing phi29 DNAP exhibits high processivity and robust strand displacement activity at 30ºC, its activity and stability decline significantly at elevated temperatures. This limitation restricts its applications, particularly in cases requiring specific thermal conditions to optimize specificity or to denature complex secondary structures in DNA templates (Kamtekar *et al.* 2004, de Vega *et al.* 2010, Manrao *et al.* 2012).

Therefore, we employed VenusREM to enhance the activity of phi29 DNAP at a higher temperature (i.e. 42ºC). We selected the C3 mutant as the template for further improvement, which is a phi29 DNAP mutant obtained through earlier directed evolution with high thermostability. However, it exhibits low ssDNA yield during *in vitro* amplification at 42ºC (Fig. 3d) and requires further enhancement (Povilaitis *et al.* 2016). We designed 10 single-site mutations from C3 and evaluated their RCA activity at 42ºC using an ssDNA template (Fig. 3c). The average results from three independent repetitions show that eight of these mutants exhibit a significant activity enhancement over C3 with an over three-fold increase (Fig. 3d). The top-performing mutant, phi29 DNAP${}^{Y449G}$, achieved a 6.5-fold increase in activity. Additionally, we assessed the thermostability of the mutants using DSF, observing a clear increase in *T*${}_{m}$ values in three of the eight active positive mutants (Fig. 3e). Notably, in directed evolution, there is generally a tradeoff between activity and stability. However, the phi29 DNAP mutants obtained by VenusREM, such as phi29 DNAP${}^{L567E}$ and phi29 DNAP${}^{S551L}$, demonstrate simultaneous improvements in both assays, presenting a new possibility for designing high-performance variants.

## 4 Discussion and conclusion

Protein engineering is a central topic in synthetic biology, where novel mutants are typically identified through rational design, machine learning, or pretrained deep neural networks. Recent advances in pretrained deep learning methods have shown remarkable success in discovering favorable mutations. These protein embedding algorithms often benefit from incorporating multiple protein modalities to generate more expressive vector representations. For instance, as highlighted by Notin *et al.* (2024) and Tan *et al.* (2023), structure-aware models are generally more effective at enhancing protein stability and binding, whereas sequence-centric models excel at improving enzymatic activity. However, the potential insights offered by evolutionary information remain underexplored. Conserved regions in proteins, for example, are often poor candidates for mutations, and AA conservations are typically derived from evolutionary analyses of homologous sequence data. Furthermore, while structural models often rely on predicted 3D structures in the absence of crystallographic data, these predictions may contain inaccuracies. Homologous sequences can mitigate this issue by providing explicit coevolutionary relationships between residue pairs (Supplementary Figs S1 and S2). Unlike existing approaches, we propose a simple yet flexible method for seamlessly integrating evolutionary information from homologous sequences into pretrained models.

On the evaluation side, the current framework heavily relies on high-throughput datasets such as ProteinGym, which facilitate statistically significant comparisons of zero-shot models without incurring experimental costs. However, high-throughput experiments often suffer from inaccuracies and are heavily biased toward negative mutants, raising concerns about the practical utility of top-performing models. To address this limitation, we extract low-throughput experimental data and establish a multidimensional post hoc analysis scheme. This approach provides a validation mechanism similar to wet-lab experiments but without the need for new experimental data. It allows researchers without access to biological experimental facilities to independently assess their models and enables direct comparisons between different deep learning approaches. In this study, we further demonstrate the practical utility of our method by selecting an important enzyme with specific modification requirements, performing single-site mutations, and experimentally validating the results. Our experimental and analytical findings indicate that the proposed model significantly outperforms existing methods on large-scale datasets, exhibits greater reliability on small-scale experimental data, and identifies novel mutants with improved activity and stability in real-world applications.

## Supplementary Material

btaf189_Supplementary_Data

## Data Availability

The data underlying this article are available in GitHub repository named VenusREM, at https://github.com/ai4protein/VenusREM.
