# Supplementary material for: From high-throughput evaluation to wet-lab studies: advancing mutation effect prediction with a retrieval-enhanced model
Source: Bioinformatics. 2025 Jul 15;41(Suppl 1):i401–9. doi: 10.1093/bioinformatics/btaf189 (PMC12261477; doi:10.1093/bioinformatics/btaf189)
Supplement: btaf189_Supplementary_Data [file btaf189_supplementary_data.pdf]

Table S1. List of baseline models for in-silico comparison and their availability.

Model Name	Model Type	Implementation
ProSST	Hybrid - Structure & PLM	https://github.com/ai4protein/ProSST
PoET	Hybrid - Alignment & PLM	https://github.com/OpenProteinAI/PoET
VesspaG	Protein language model	https://github.com/JSchlensok/VespaG
SaProt	Hybrid - Structure & PLM	https://github.com/westlake-repl/SaProt
TranceptEVE	Hybrid - Alignment & PLM	https://github.com/OATML-Markslab/Tranception
GEMME	Alignment-based model	https://hub.docker.com/r/elodielaine/gemme
ProtSSN	Hybrid - Structure & PLM	https://github.com/tyang816/ProtSSN
EVE	Alignment-based model	https://github.com/OATML-Markslab/EVE
VESPA	Protein language model	https://github.com/Rostlab/VESPA
Tranception	Hybrid - Alignment & PLM	https://github.com/OATML-Markslab/Tranception
MSA Trans	Hybrid - Alignment & PLM	https://github.com/facebookresearch/esm
ESM-IF1	Inverse folding model	https://github.com/facebookresearch/esm
DeepSequence	Alignment-based model	https://github.com/debbiemarkslab/EVcouplings
ESM2	Protein language model	https://github.com/facebookresearch/esm
ESM-1v	Protein language model	https://github.com/facebookresearch/esm
MIF-ST	Hybrid - Structure & PLM	https://github.com/microsoft/protein-sequence-models
EVmutation	Alignment-based model	https://github.com/debbiemarkslab/EVcouplings
ESM-1b	Protein language model	https://github.com/facebookresearch/esm

Table S2. Summary of homology retrieval tools and statistics for the retrieved phonological sequences for assays in ProteinGym.

			**Statistics**				
**Strategy**	**Description**	**Format**	maximum #	minimum #	median #	average #	standard deviation
EVcouplings[3]	sequence homologs	.a2m	1,879,223	44	40,154	128,297	235,939
ColabFold[4]	sequence homologs	.a3m	26,534	38	8,292	8,651	6,698
Foldseek[5]	structure homologs	.json	7,773	0	3,606	3,529	1,986

Table S3. List of the nine source databases for structural homologs retrieval.

**Database**	**Source**
mgnify_esm30	https://www.ebi.ac.uk/metagenomics
afdb50	https://alphafold.ebi.ac.uk/
afdb-proteome	https://alphafold.ebi.ac.uk/
cath50	https://www.cathdb.info/
pdb100	https://foldseek.steineggerlab.workers.dev/
afdb-swissprot	https://alphafold.ebi.ac.uk/
gmgcl_id	https://gmgc.embl.de/
bfvd	https://bfvd.steineggerlab.workers.dev/

Experimental Procedures for phi29 DNAP

Construction of phi29 DNAP expression plasmid

The gene of phi29 DNA polymerase and its variants genes were synthesized by Sangon Biotech (Shanghai, China) after codon-optimized. These genes were subsequently cloned into the pET28(a) plasmid, which features an N-terminal His-tag for protein purification.

Protein expression of phi29 DNAP

The expression plasmid was transformed into *E. coli* BL21(DE3). A 30 mL seed culture was initially cultivated at 37 °C in LB medium supplemented with 50 μg/ml kanamycin, which was then transferred to 500 mL of LB medium containing the same concentration of kanamycin in a shaker flask. The cultures were incubated at 37 °C until the OD600 reached 1.0, and protein expression was then induced by the addition of isopropyl-D-thiogalactopyranoside (IPTG) to a final concentration of 0.5 mM, followed by incubation for 16 hours at 25 °C.

Protein purification of phi29 DNAP

Cells were harvested by centrifugation for 30 minutes at 4,000 rpm, and the resulting pellets were collected for subsequent purification. The cell pellets were then resuspended in lysis buffer (25 mM Tris-HCl, 500 mM NaCl, pH 7.4) and disrupted via ultrasonication (Scientz, China). The lysates were centrifuged at 12,000 rpm for 30 minutes at 4 °C, and the supernatants were subjected to Ni-NTA affinity purification using an elution buffer composed of 25 mM Tris-HCl, 500 mM NaCl, and 250 mM imidazole (pH 7.4). Following purification, the protein was desalted with lysis buffer through ultrafiltration. The protein-containing fractions were then flash-frozen at -20 °C in a storage buffer consisting of 25 mM Tris-HCl (pH 7.4), 200 mM NaCl, and 20% glycerol.

Rolling circle replication assay of phi29 DNA polymerase

We assessed the RCA reaction to simulate the circular template amplification activity of phi29 DNA polymerase. Rolling circle amplification (RCA) was conducted using the M13mp18 single-stranded DNA (ssDNA) template (NEB, United States) in a 20 μL reaction volume. The reaction mixture included 2 μL of M13mp18 template (0.2 μg/μL), 2 μL of 10× RCA buffer (Thermo Fisher, United States), 2 μL of phi29 DNA polymerase (100 ng/μL), 4.8 μL of primer mix (2.4 μM final concentration), 0.8 μL of dNTPs (10 mM each), and 8.4 μL of DEPC-treated water (LABTOP BIO, China). The RCA was performed at 42°C for 10 minutes, followed by a denaturation step at 65°C for 10 minutes. DNA concentrations were subsequently

Table S4. Spearman's ρ of mutation effect prediction (substitution) by zero-shot predictions on **ProteinGym** of different MSA depth and taxon.

model	MSA Depth			Taxon			
	Low	Medium	High	Human	Eukaryote	Prokaryote	Virus
VenusREM	**0.504**	**0.525**	**0.572**	**0.529**	**0.590**	**0.538**	**0.492**
ProSST	**0.473**	**0.511**	**0.578**	**0.516**	**0.573**	**0.549**	**0.454**
PoET	**0.488**	**0.472**	0.515	**0.482**	**0.541**	**0.564**	**0.491**
VespaG	0.443	0.469	0.513	0.474	0.532	0.484	0.421
SaProt	0.395	0.450	**0.545**	0.476	0.523	0.524	0.300
TranceptEVE	0.451	0.467	0.492	0.471	0.498	0.473	0.453
GEMME	0.455	0.470	0.497	0.468	0.510	0.473	0.469
ProtSSN	0.401	0.458	0.522	0.469	0.518	0.505	0.356
EVE	0.425	0.453	0.481	0.453	0.487	0.468	0.428
VESPA	0.427	0.455	0.484	0.438	0.492	0.490	0.432
Tranception	0.432	0.438	0.473	0.453	0.483	0.431	0.432
MSA Trans	0.393	0.435	0.473	0.427	0.491	0.451	0.390
ESM-if1	0.300	0.431	0.544	0.415	0.497	0.507	0.374
DeepSequence	0.383	0.428	0.473	0.442	0.469	0.460	0.344
ESM2	0.335	0.406	0.515	0.456	0.471	0.476	0.238
ESM-1v	0.326	0.405	0.499	0.456	0.446	0.432	0.279
MIF-ST	0.376	0.403	0.455	0.398	0.415	0.462	0.397
EVmutation	0.403	0.421	0.410	0.409	0.444	0.422	0.388
ESM-1b	0.350	0.398	0.482	0.434	0.475	0.455	0.241

† The top three scores are highlighted by **First**, **Second**, and **Third**.

Table S5. Performance of retrieval ratios for different methods on 10% samples of each assay of ProteinGym.

Retrieval	**0.1**	**0.2**	**0.3**	**0.4**	**0.5**	**0.6**	**0.7**	**0.8**	**0.9**
`EVCouplings`	0.516	0.518	0.526	0.521	0.526	0.532	0.529	0.541	0.529
`ColabFold`	0.516	0.517	0.525	0.519	0.522	0.527	0.524	0.534	0.521
`Foldseek`	0.515	0.517	0.524	0.518	0.521	0.527	0.522	0.533	0.526
`EVCouplings+Foldseek`	0.516	0.519	0.528	0.525	0.532	0.536	0.527	0.506	0.430

measured using the ssDNA Assay Kit (YEASEN, China) with a Qubit4 fluorometer (Thermo Fisher, United States).

Differential Scanning Fluorimetry (DSF)

The T_m values were measured by DSF with the Protein Thermal Shift Dye Kit (Thermo Fisher, U.S.A.). To prepare the reaction mixture, 1.0 μL of SYPRO Orange Dye (SUPELCO, U.S.A.) was added to 49 μL of lysis buffer (25 mM Tris-HCl, 500 mM NaCl, pH 7.5). Then 1 μL of the diluted dye was combined with 19 μL of a 0.2 mg/mL protein solution. DSF experiments were conducted by the LightCycler 480 Instrument II (Roche, U.S.A.). The reaction mixture was initialized at 25 °C and gradually heated to 99 °C at a rate of 0.1 °C s^{-1}. Data analysis was performed by Protein Thermal Shift.

Baseline Methods

Our comparison on ProteinGym encompasses a range of state-of-the-art models, including sequence-based models, sequence-structure models, alignment-based models, and inverse folding models. The reproduction and ranking of these models were officially conducted and made publicly available. For the case study comparisons, we specifically focus on the leading sequence-based and sequence-structure models, such as `SaProt`, `ProtSSN`, `ESM2`, and `MIF-ST`. Detailed implementations of the baselines are provided in Table S1.

Supplementary Results on ProteinGym

The detailed Spearman scores can be found in Figs S3-S7.

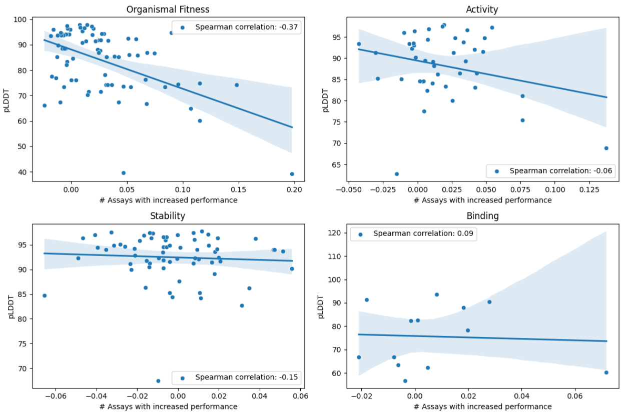

Fig. S1. Spearman score difference between with and without retrieval by pLDDT.

We also evaluated each model's performance on **ProteinGym** across different MSA depths and taxonomic groups, as shown in Table S4. VenusREM demonstrated notable improvements across all categories, particularly in scenarios with limited MSA depth and in traditionally challenging categories, such as humans and viruses.

We held out 10% of mutants from each assay as a validation set to evaluate the performance of different retrieval methods and the generalizability of retrieval factors. The detailed results are presented in Table S5. Notably, the optimal performance is

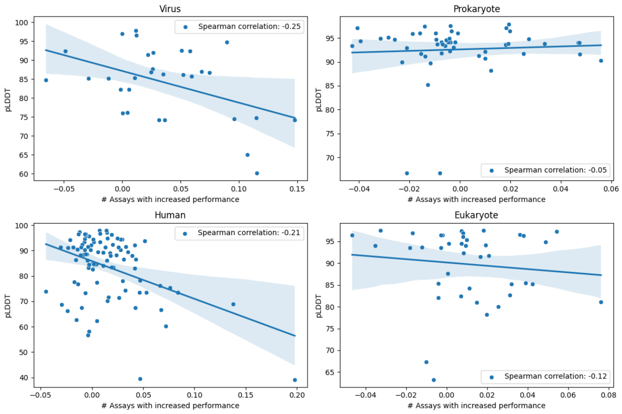

Fig. S2. Spearman score difference between with and without retrieval by taxon.

achieved when using the official `.a2m` homologous sequence with $\alpha = 0.8$.

With homologous information and predicted protein structures being simultaneously input into the model, we aim to determine whether the explicit addition of homologous information can compensate for deficiencies in protein structure quality. As shown in Fig. S1 and S2, a generally negative correlation between `pLDDT` and score improvement is observed across categories, except for Binding (likely due to a limited number of data points). This indicates that the lower the structural quality, the more effective the homologous information becomes.

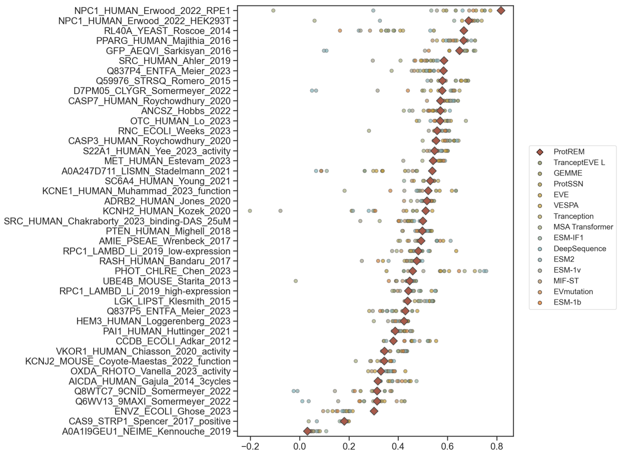

Fig. S3. Spearman's correlation of individual assay - Activity

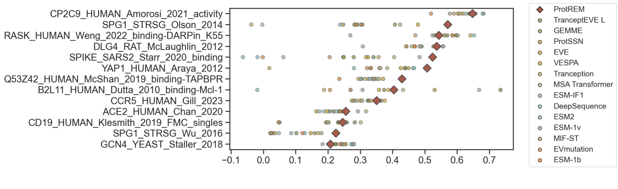

Fig. S4. Spearman's correlation of individual assay - Binding

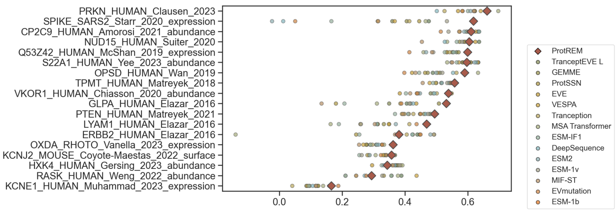

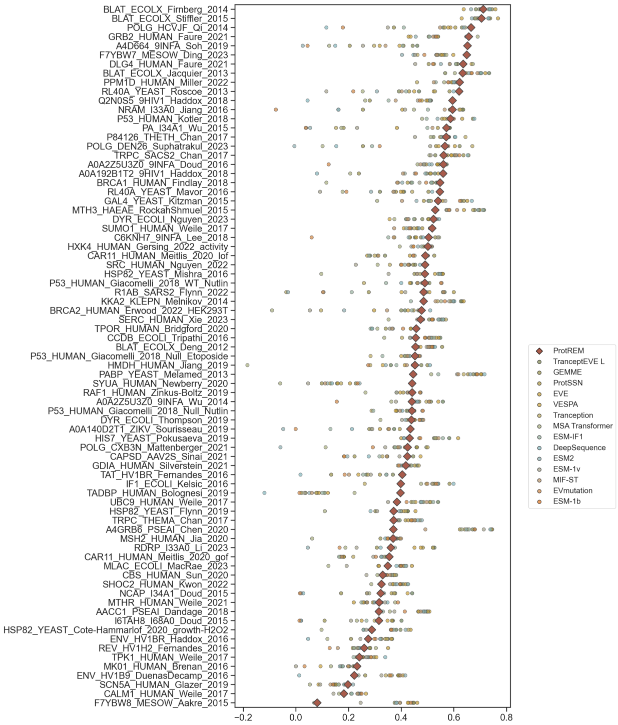

Fig. S6. Spearman's correlation of individual assay - Organismal Fitness

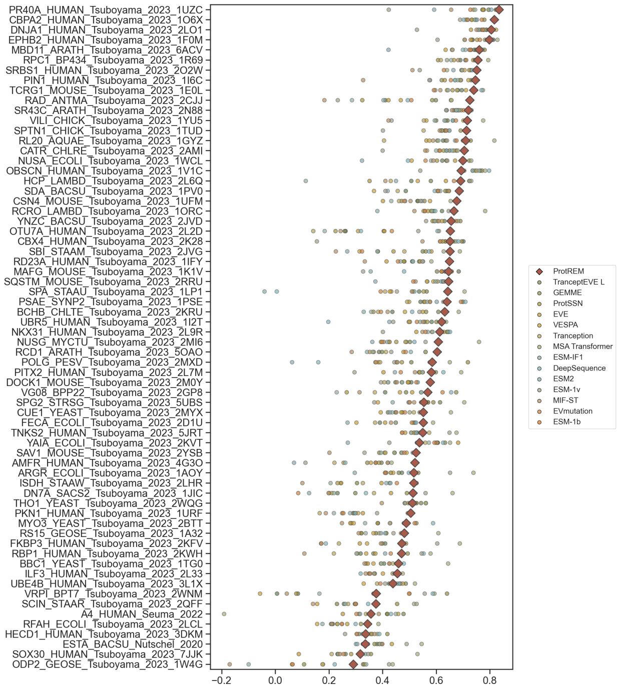

Fig. S7. Spearman's correlation of individual assay - Stability
